# Non-Invasive Quantification of Cartilage Using a Novel *In Vivo* Bioluminescent Reporter Mouse

**DOI:** 10.1371/journal.pone.0130564

**Published:** 2015-07-07

**Authors:** Sarah E. Mailhiot, Donald L. Zignego, Justin R. Prigge, Ella R. Wardwell, Edward E. Schmidt, Ronald K. June

**Affiliations:** 1 Molecular Biosciences Program, Montana State University, Bozeman, MT, United States of America; 2 Department of Microbiology and Immunology, Montana State University, Bozeman, MT, United States of America; 3 Department of Mechanical and Industrial Engineering, Montana State University, Bozeman, MT, United States of America; 4 Department of Cell Biology and Neuroscience, Affiliate Faculty, Montana State University, Bozeman, MT, United States of America; Stony Brook University, UNITED STATES

## Abstract

Mouse models are common tools for examining post-traumatic osteoarthritis (OA), which involves cartilage deterioration following injury or stress. One challenge to current mouse models is longitudinal monitoring of the cartilage deterioration *in vivo *in the same mouse during an experiment. The objective of this study was to assess the feasibility for using a novel transgenic mouse for non-invasive quantification of cartilage. Chondrocytes are defined by expression of the matrix protein aggrecan, and we developed a novel mouse containing a reporter luciferase cassette under the inducible control of the endogenous aggrecan promoter. We generated these mice by crossing a Cre-dependent luciferase reporter allele with an aggrecan creERT2 knockin allele. The advantage of this design is that the targeted knockin retains the intact endogenous aggrecan locus and expresses the tamoxifen-inducible CreERT2 protein from a second IRES-driven open reading frame. These mice display bioluminescence in the joints, tail, and trachea, consistent with patterns of aggrecan expression. To evaluate this mouse as a technology for non-invasive quantification of cartilage loss, we characterized the relationship between loss of bioluminescence and loss of cartilage after induction with (i) *ex vivo* collagenase digestion, (ii) an *in vivo* OA model utilizing treadmill running, and (iii) age. *Ex vivo* experiments revealed that collagenase digestion of the femur reduced both luciferase signal intensity and pixel area, demonstrating a link between cartilage degradation and bioluminescence. In an *in vivo* model of experimental OA, we found decreased bioluminescent signal and pixel area, which correlated with pathological disease. We detected a decrease in both bioluminescent signal intensity and area with natural aging from 2 to 13 months of age. These results indicate that the bioluminescent signal from this mouse may be used as a non-invasive quantitative measure of cartilage. Future studies may use this reporter mouse to advance basic and preclinical studies of murine experimental OA with applications in synovial joint biology, disease pathogenesis, and drug delivery.

## Introduction

Osteoarthritis (OA) affects the majority of the human population older than age 65, making it the most common degenerative joint disease [1]. The development of pharmacological treatment for OA is challenging due to difficulties with effective intra-joint drug delivery [2,3], the existence of relatively few validated therapeutic targets for treatment [4], and concerns about assessment or relevance of small animal models [5].

Mouse models for OA are hindered by a lack of standardized grading scales [6] and imaging techniques [7]. Murine experimental arthritis can be induced by a variety of methods including proteolytic enzyme injection [8], chemical-induced cell death [9], surgical destabilization [10,11], and applied external loading [12]. Each of these approaches mimics pathology of human disease including cartilage degeneration, chondrocyte death, and cartilage deterioration. However repeated quantification of cartilage deterioration within the same animal is not possible with current technology. Thus, existing analytical techniques can only be performed post-mortem, and therefore prevent longitudinal observation of the same mouse in response to potential therapeutic treatments. This study focused on improving quantification of the progression of OA in mouse models through the use of *in vivo* bioluminescence imaging.

Current approaches for quantifying the extent of cartilage deterioration in murine experimental arthritis include histopathological grading scales [6,13] and assessment via micro-CT imaging [14–16]. Histopathological grading scales such as the Osteoarthritis Research Society International (OARSI) guidelines often involve staining of fixed and decalcified joint sections (*e*.*g*. saffranin-O/fast green on formalin fixed, paraffin-embedded coronal sections), which are evaluated by blinded observers who assign discrete values to the extent of cartilage damage both along the joint surface and in the direction of the subchondral bone. The limitations of quantifying cartilage damage using this method have been well documented [6] and include (i) limited ability to discriminate small differences and subtle phenotypes, (ii) inter-observer variability, and (iii) required euthanasia of the experimental animal. Micro-CT is a promising technique which can provide high-resolution and three-dimensional quantification of cartilage within a murine synovial joint [14–16]. However, the accumulated radiation dose delivered to the animals complicates interpretation of studies involving the repeated analyses of the same individuals [17]. *In vivo* imaging has examined the activity of NF-κB and MMPs [18–20], but these methods do not provide quantitative information about articular cartilage.

The objective of this study was to assess the feasibility for using a novel transgenic mouse for non-invasive quantification of cartilage. Because the structural matrix protein aggrecan is a well-characterized marker of the chondrocyte phenotype, we crossed mice containing an inducible Cre Recombinase nondisruptively knocked into the endogenous murine *aggrecan* locus [21] with mice containing a floxed luciferase allele [22]. The mice display bioluminescence specifically in tissues expressing aggrecan at the time of induction (*e*.*g*. knee joints, intervertebral discs, etc.) which can be monitored non-invasively using quantitative *in vivo* bioluminescence imaging. We here show that this mouse produces bioluminescence from aggrecan-expressing tissues including articular cartilage. Furthermore, we observed decreases in the bioluminescent signal which correlated with cartilage changes in OA models. The advantage of this genetic strategy is the ability to time-stamp aggrecan-expressing chondrocytes prior to initiation of an OA model which can induce variation in aggrecan expression. This mouse may be useful for (1) lineage-tracing studies of chondrocyte biology, (2) quantification of OA progression in experimental OA studies, and (3) longitudinal studies examining the efficacy of therapeutic interventions in preventing cartilage damage or inducing cartilage repair.

## Methods

### Animals and Animal Care

**Fig 1:**
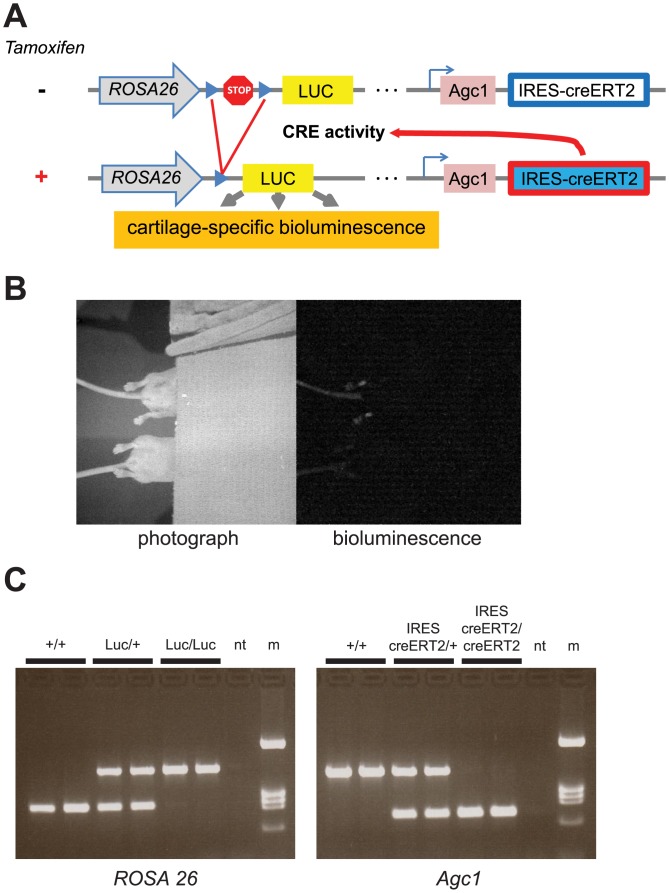
Genetic strategy for transgenic mouse with chondrocyte-specific bioluminescence. (A) Schematic of genetic construction. (B) Representative photograph (left) and quantitative bioluminescence image (right). Note that bioluminescence in the trachea was filtered using a surgical towel. (C) Representative genotyping results. The transgenic reporter mouse utilizes an IRES-driven inducible Cre Recombinase (Cre-ERT2) cistron inserted into the 3’ untranslated region of the wild-type aggrecan locus Agc1 [21]. Upon Cre activation via tamoxifen, bioluminescence reporting is induced in aggrecan-producing cells (*e*.*g*. chondrocytes) via administration of luciferin, which is detectable via *in vivo* bioluminescence imaging. Mouse genotypes were inferred via PCR analysis using custom-designed primers at either the ROSA 26 locus for luciferase (panel C, left) or the Agc1 locus for Cre-ERT2 (panel C, right). nt = no template control; m = DNA size marker (sizes from top to bottom are 1380, 517, 456, 396, and 247 bp).

All animal studies adhered to the guidelines prescribed by the US National Institutes of Health and the 8^th^ Edition of the Guide for the Care and Use of Animals, and were approved by the Montana State University Institutional Animal Care and Use Committee (Protocol Number: 2012–16). Mice were housed 3–5 per cage with 12 hour light-dark cycles and *ad libitum* access to food and water. LoxP-STOP-LoxP-Luciferase reporter mice (LSL-Luc) were obtained from Jackson Labs (Stock Number: 005125) on a C57/BL6 background [22]. LSL-Luc mice are capable of expressing luciferase in Cre-expressing tissues. Aggrecan-Cre-ER^T2^ mice (Agc-Cre) were obtained from the DeCrombugge Laboratory [21] on a 129S6 x C57/BL6 background. Agc-Cre mice contain the Cre-ER^T2^ cistron inserted into the 3’ UTR of the aggrecan locus. This cassette contains an internal ribosomal entry sequence (IRES) to drive translation of the recombinase from the bicistronic mRNA in all cells that express the endogenous aggrecan gene. This yields inducible Cre Recombinase expression only in aggrecan-expressing tissues (*e*.*g*. articular cartilage). Experimental mice were obtained by breeding LSL-Luc mice with Agc-Cre mice (Fig 1A and 1B).

### Genotyping

Genotypes of all mice were determined by PCR amplification of genomic tail DNA as follows. The ROSA26+ and Luc alleles were amplified in a single reaction containing the following primers: R26-forward, 5’ cctcgtgatctgcaactccagtc 3’; R26-reverse, 5’ gggagaaatggatatgaagtactgg 3’; Luc-forward, 5’ taccagggatttcagtcgatgtac 3’; and Luc-reverse, 5’ tcgtcttcgtcccagtaagctatg 3’. This primer set gives the following allele-specific product sizes: R26+, 425 bp; R26Luc, 819 bp. The Agc1 + and IRES-creERT2 alleles were amplified in a single reaction containing the following primers: Agc-forward, 5’ gagcctcgaatcacctgcacagac 3’; Agc-reverse, 5’ ctaaactcagtccacgggttacagt 3’; Cre-forward, 5’ aggttcgcaagaacctgatggacatg 3’; and Cre-reverse, 5’ cttttcggatccgccgcataa 3’. This primer set gives the following allele-specific product sizes: Agc+, 719 bp; AgcIRES-creERT2, 303 bp. PCR products were analyzed via gel electrophoresis and visualized with ethidium bromide staining (Fig 1C).

### Luciferase Induction, Imaging, and Image Processing

Cre-ERT2 activity was induced via subcutaneous scruff injection of tamoxifen free base (TFB, 10–15 mg/mL in vegetable oil) [23]. To optimize induction, we compared different administration protocols. TFB was administered daily for 1–5 days at 2 mg/day for male mice or 1.5 mg/day for female mice, based on average weight.

Following a 3 day rest period, *in vivo* imaging was performed as follows for both groups. Mice were anesthetized using isoflourane (2–3% v/v at 2.5 mL/min in oxygen) and exogenous D-luciferin potassium salt, a substrate required for production of bioluminescence in the presence of luciferase, was then administered at a dose of 15 mg/mL subcutaneous scruff injection for a total dose of 2.25 mg in 15 μl. Imaging was performed with the abdominal side of the mouse facing the detector and utilizing black paper to filter signal from the trachea and focus the examination of the unshaved joint of interest (*i*.*e*. knee joints) in a single image. Prior to quantitative bioluminescence imaging, photographs were obtained using 15 second exposures. Bioluminescence images were obtained under anesthesia with 15 minute exposures for 0–60 minutes following luciferin administration using a Kodak ImageStation 2000MM multi-modal *in vivo* imaging system. While longer image exposures have been reported in the literature, we selected 15 minutes based on pilot data obtained comparing 10 minute and 15 minute exposures and optimization for imaging equipment.

Female mice aged 2 months and older were used for this study. Preliminary data showed that female mice produce bioluminescence. Ages of 2 months were selected based on previous studies of murine osteoarthritis [24–26].

Raw 16-bit TIFF images were analyzed using Matlab with the following methods. Images were contrasted using the imtool function. Complements were taken of the image and thresholded using a gray threshold. The image was then converted to black and white and filtered using a median filter with a size of 16 pixels [27]. A region of interest was selected around each knee using imtool. The total pixel intensity was calculated as the sum of pixel values in the region. The normalized pixel intensity was calculated as the total pixel intensity divided by the baseline average for that knee. The area of pixel intensity was calculated as the number of pixels with a value greater than a threshold of 20,000 on a 16 bit scale (65,563 maximum pixel value) in the region of interest. Area calculations were performed over the entire region of interest such that the number of pixels represented the location of the signal in two dimensional space.

### *Ex Vivo* Digestion Assay

To assess the ability of the bioluminescent signal to quantify relevant pathophysiological changes within the joint, and thereby be a useful metric for measuring cartilage deterioration, harvested joint tissue was digested enzymatically during repeated bioluminescent imaging. First, mice were induced with 5 days of tamoxifen followed by a 5 day rest period. Immediately after euthanization via CO_2_ inhalation, murine knee joints were harvested. Under a dissecting microscope, non-cartilaginous tissues (*e*.*g*. joint capsule, ligament, menisci, etc.) were removed with a scalpel prior. The distal femoral heads (n = 4) were subjected to collagenase digestion.

Harvested tissues were immersed in collagenase (Type IV, Sigma-Aldrich, 0.5 mg/mL in serum-free Dulbecco’s Modified Eagle’s Medium without antibiotics) for 0–12 hours at 37°C. At each timepoint, tissues were removed from digestion and washed twice with PBS. Samples were then immersed in D-luciferin potassium salt solution (5 mg/mL in PBS) in 96 well plates for bioluminescent imaging. Images were taken with a 15 minute exposure at each time point.

### *In Vivo* Models: Treadmill Running and Surgical Destabilization

To test the ability to detect bioluminescence changes in an *in vivo* OA model, transgenic mice were run on a treadmill at a maximum speed of 34 cm/s and 15 degree incline for up to 20 minutes based on a prior exercise-induced model of OA and joint fibrosis [28]. Cartilage-specific bioluminescence was induced by 5 days of tamoxifen followed by 5 days of rest. The mice were initially trained for 10 days during which the speed was gradually increased over the first 10 minutes of each session. The mice were then run for 5 days to further cartilage deterioration. Subsequently, mice were imaged periodically for 51 days prior to sacrifice via CO_2_ inhalation for histological studies.

In an additional *in vivo* model, we performed surgical destabilization in conjunction with treadmill running. Mice were randomized into 2 groups: sham unexercised controls (CTRL) and surgically destabilized exercised (SDE) mice (n = 3 each). Cre activity was induced by 5 days of serial tamoxifen administration via subcutaneous scruff injection of 1.2 mg/day. SDE mice were then trained in treadmill running for 10 days as described above. In each group, mice were rendered unconscious via isoflourane and a medial incision was created on the left knee. For the SDE group, the medial collateral ligament was transected and the medial meniscus was removed using previous methods [11]. Incisions were sutured, and mice were administered buponepherine at 0.5 mg/kg. Following a 3 day rest period, the SDE group was exercised for an additional 15 days prior to euthanization. Bioluminescence imaging was performed every 2 days following bouts of treadmill running.

### Histology

At appropriate timepoints, mice were euthanized via CO_2_ inhalation. Knee joints were dissected and frozen in cryosectioning media (O.C.T. Compound, Sakura Finetek USA, Inc., Torrance, CA,USA). Samples were then cut in 20 micron sections and placed on transparent tape (Cryofilm Type 2C, Section-Lab, Hiroshima, Japan [29]). The samples were then stained with 0.04% Toluidine Blue O for 5 minutes, washed in tap water, then stained in 0.1% Fast Green FCF for 2 minutes. To quantify cell numbers, samples were stained with 0.1% Mayers Hematoxylin for 10 minutes, washed in tap water for 5 min, then rinsed 10 times 0.5% Eosin Y. The samples were imaged at 4x and 10x objective magnification.

### Experimental Design, Analysis, and Statistics

The experimental unit for these studies was the region of interest defined in a single mouse knee. To assess the relationship between the quantitative bioluminescence imaging outputs, Pearson correlations examined the relationship between pixel area and total pixel intensity (318 images). To determine the effect of the number of days of serial tamoxifen injections on bioluminescence, total pixel intensity and area were analyzed after 1, 3, and 5 days of injection using a single factor analysis of variance (ANOVA, n = 8 knees). To determine the effect of age on bioluminescence, bioluminescence was induced in mice at 2 months of age and 5 months of age (n = 10 mice) and was analyzed using a single factor ANOVA. To determine if bioluminescence decreased as a function of digestion time in collagenase, images were recorded every 2 hours prior to correlation analysis between time and total pixel intensity (n = 5 femurs). To determine the effect of *in vivo* treadmill running, bioluminescence was recorded after 5 days of running (n = 5 mice, one knee per mouse). The Pearson correlation coefficient was calculated to assess the relationships between time and *in vivo* bioluminescence. All experiments except the surgical destabilization utilized a minimum of 5 mice in each group. The surgical destabilization experiment utilized n = 3 mice. Data are presented as mean ± standard error of the mean.

## Results

### Breeding and Genotyping

Aggrecan is a key structural component of cartilage that is specifically expressed in chondrocytes [30]. An aggrecan-driven tamoxifen-inducible Agc-cre transgene [21] was bred across the ubiquitously transcribed cre-dependent luciferase cassette, LSL-Luc and both transgenes were bred to homozygosity (Agc-cre/cre;LSL-Luc/Luc). PCR genotyping analysis differentiated between wild-type, homozygous, and heterozygous knockin mice (Fig 1C).

### Bioluminescence Imaging

**Fig 2:**
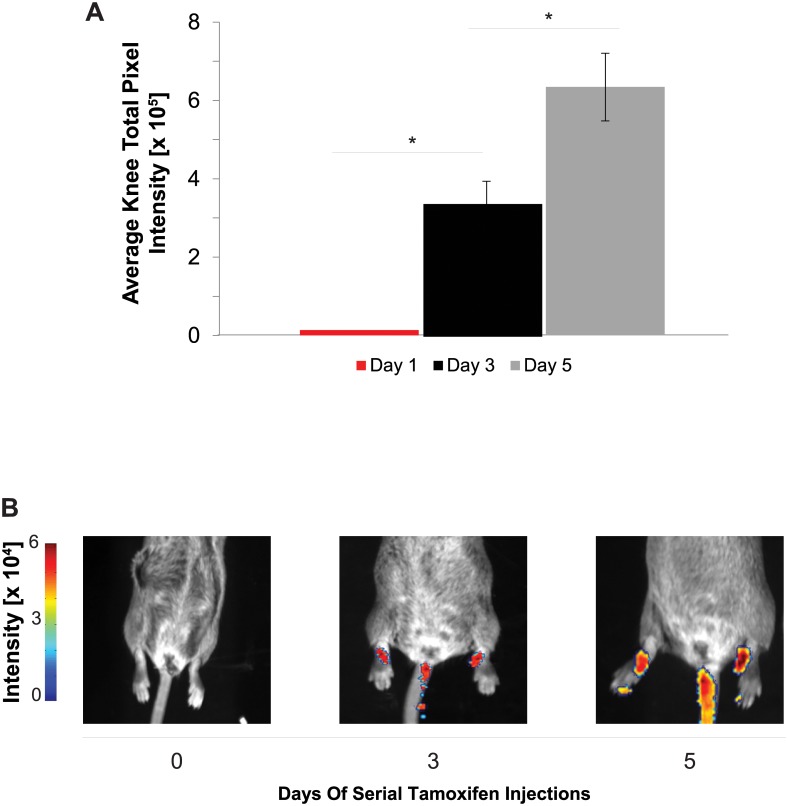
Induction of bioluminescence via tamoxifen. To examine the duration of serial injections of tamoxifen free base on joint-specific bioluminescence, we treated mice for 0–5 days via intraperitoneal injection. (A) There is an increase at 3 days (p = 0.004) and from 3 to 5 days (p = 0.01). No significant bioluminescence was detected after 1 day of Tamoxifen administration. (B) Representative bioluminescence images from 0–5 days of tamoxifen treatment.

Bioluminescence was observed to be under tight control of induction by creERT2 activity in aggrecan expressing cells including cartilage of the ankle, knee, and hip. Scruff injection of tamoxifen to activate cre for genetically encoded expression of luciferase in chondrocytes resulted in genetic induction of detectable luminescence after 3 daily injections (Fig 2, n = 4 mice). Pilot studies found substantial tracheal bioluminescence (not shown) in addition to bioluminescence within all of the joints of the lower limbs (*i*.*e*. ankle, knee, and hip). One day of tamoxifen treatment did not result in detectable bioluminescence, but three and five days of tamoxifen injections did produce detectable bioluminescence as compared to the single injection (both p < 0.01, Fig 2). Furthermore, bioluminescence was greater with five as compared to three tamoxifen injections (p = 0.01, Fig 2) indicating that increased numbers of cells activated creERT2 due to administering additional tamoxifen, which also highlights the challenges to articular cartilage drug delivery [2].

### *In Vitro* Digestion Model

**Fig 3:**
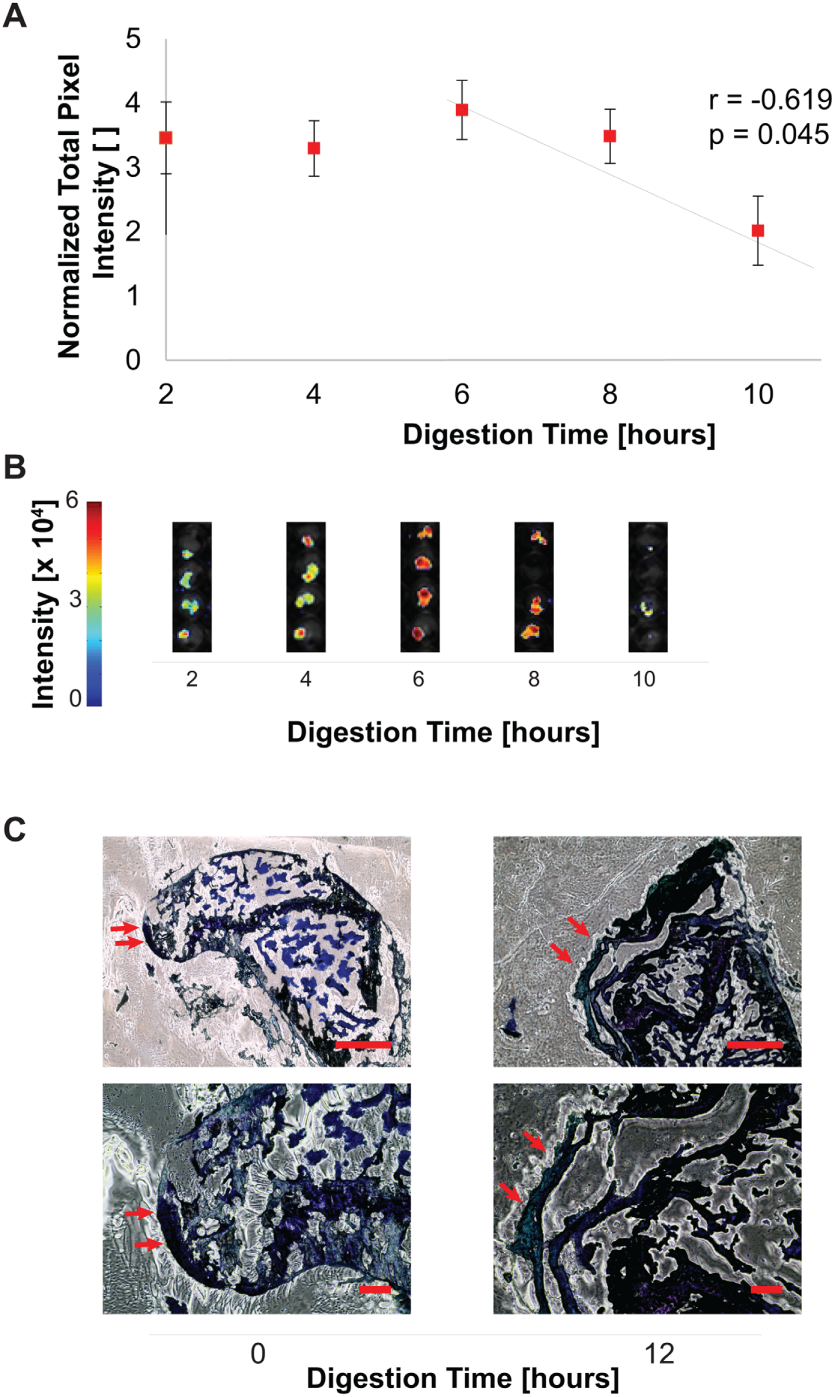
Bioluminescence decreased following *ex vivo* collagenase digestion of transgenic femora. Femora from tamoxifen treated transgenic mice were degraded in collagenase. (A) The femur showed a linear decrease (r = -0.619, p = 0.0452) after 6 hours in digestion. Signal was normalized to zero hours of digestion. (B) Whole-femur bioluminescence imaging found decreases in bioluminescence following 6 hours of digestion. Undigested femora exhibited stable bioluminescence over the experimental timecourse (not shown). (C) Terminal histology at 12 hours found a decrease in cartilage thickness correlating with the decrease in bioluminescent output. Scale bars: (4X) 200 microns (10X) 50 microns.

To assess whether enzymatic degradation such as that observed in human OA could result in decreases in bioluminescence, we subjected mouse joint tissue (n = 4 femoral heads) to collagenase digestion e*x vivo*. Collagenase digestion resulted in a decrease in bioluminescent signal from the femur with increasing digestion time after 2 hours (Fig 3). Undigested femora exhibited stable bioluminescence over the experimental timecourse (not shown). There was a negative correlation between the total pixel intensity and digestion time (r = -0.6192, p = 0.0452). Terminal histology found near-complete digestion of the articular cartilage which abrogated the luciferase signal by removing the chondrocytes. Growth plate cartilage appeared intact, indicating that the observed luciferase signal was produced by the articular cartilage (Fig 3C).

### *In Vivo* Studies

**Fig 4:**
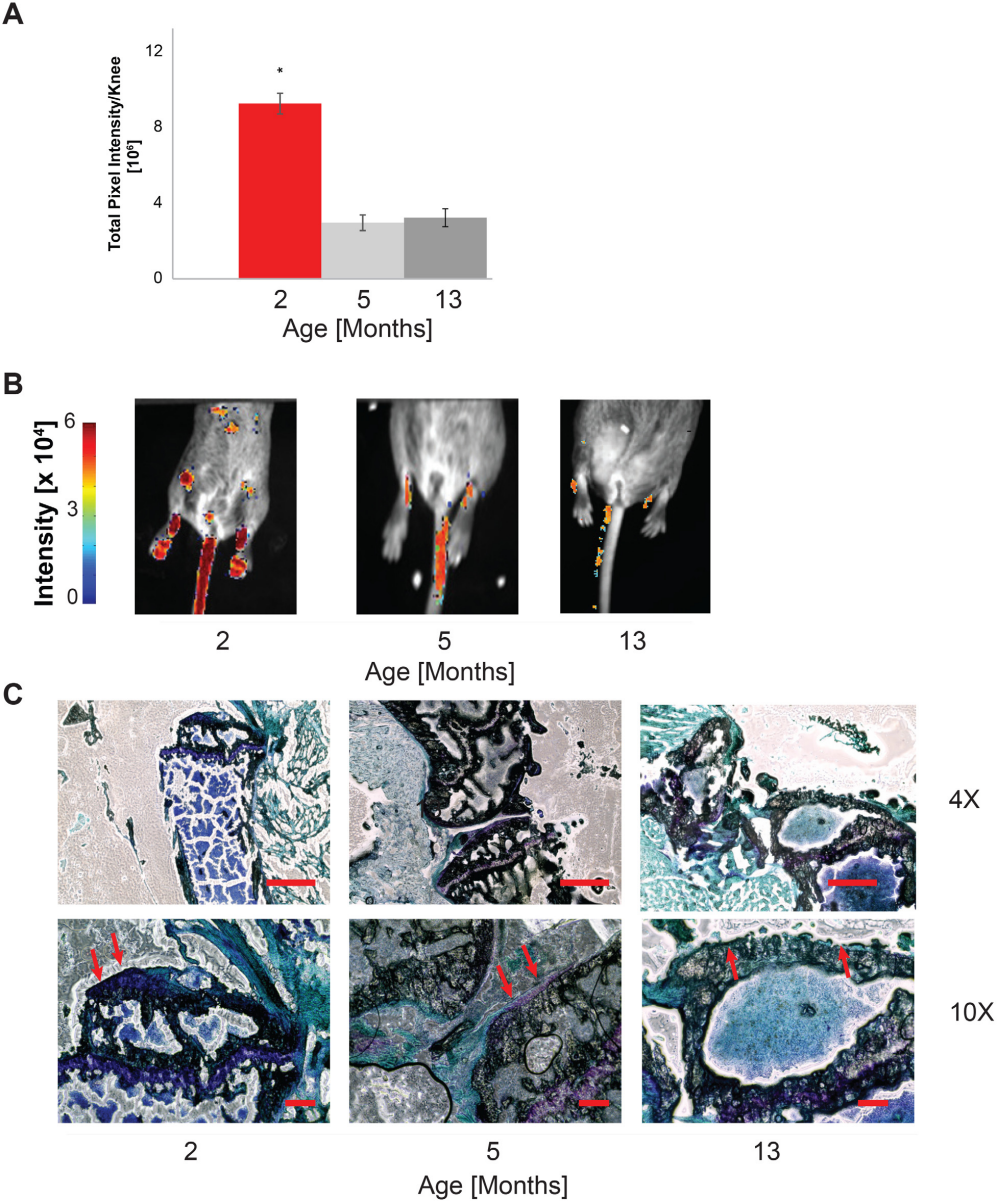
Bioluminescence decreases with aging in transgenic mice. Bioluminescence was induced in transgenic mice via 5 days of serial tamoxifen treatment, and mice were imaged at 2, 5, and 13 months of age. (A) Four times less bioluminescent signal was observed after aging from 2- to 5- and 2- to 13- months (both p < 10^-13^). (B) Representative quantitative bioluminescence images of 2-, 5-, and 13-month mice. (C) Less cartilage was found in 5- and 13-month mice when compared with 2 month mice. Scale bars: (4X) 200 microns (10X) 50 microns.

**Fig 5:**
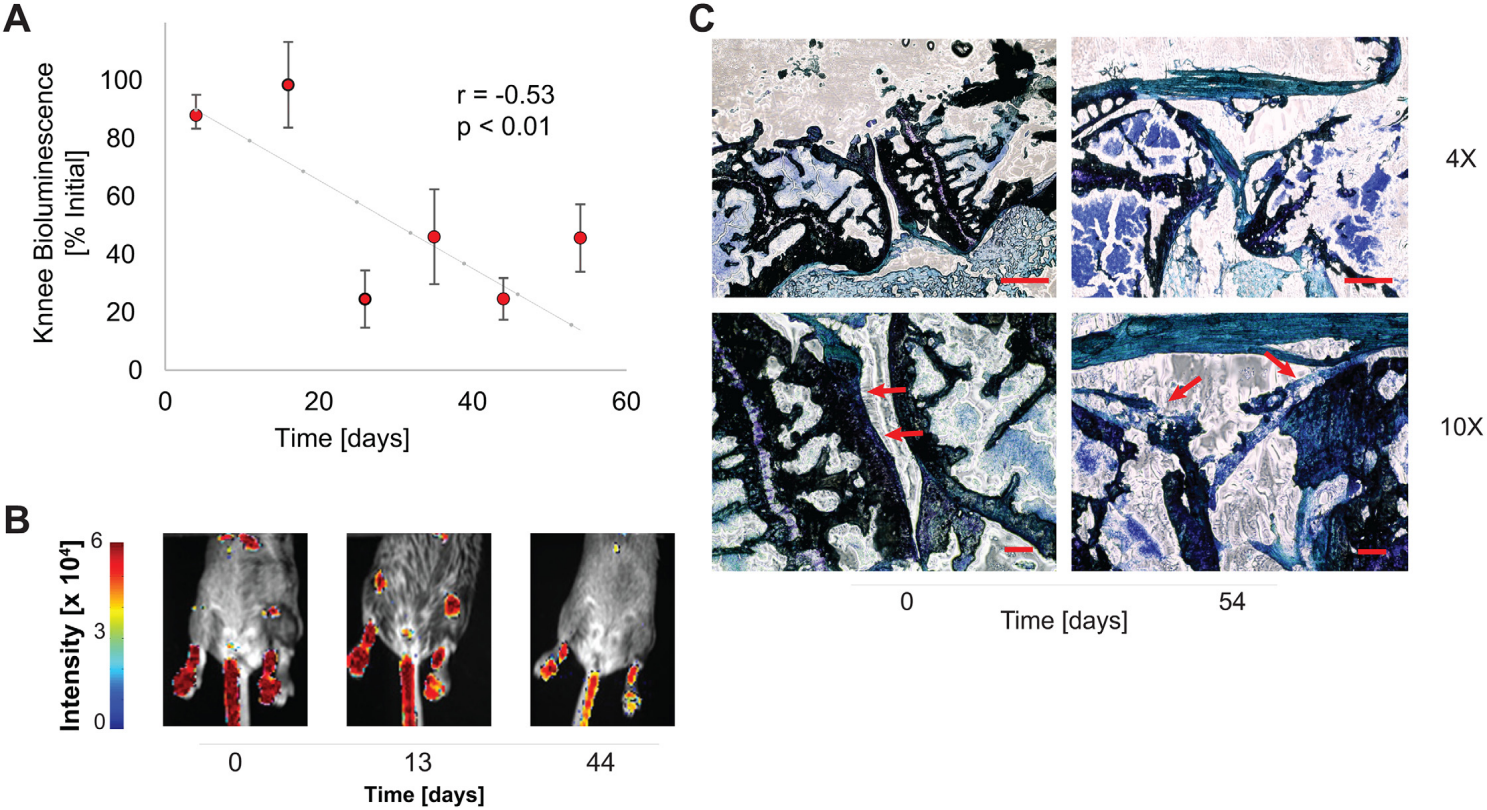
Decreased knee bioluminescence was found with increased duration in an *in vivo* deterioration model. Mice were trained and then run at maximal speeds on a treadmill for 5 days prior to bioluminescence imaging periodically over 54 days. (A) There is a negative correlation between days post running and bioluminescence (r = -0.526, p = 0.0028) (B) Representative images from timepoints of 0, 13, and 44 days. (C) Histological examination found thinner and deteriorated cartilage after 54 days compared with day 0. Scale bars: (4X) 200 microns (10X) 50 microns.

**Fig 6:**
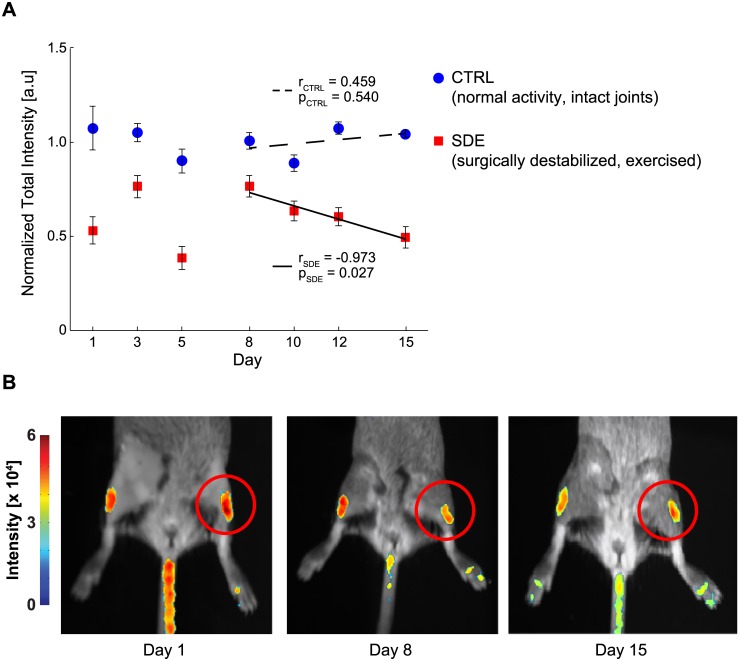
Knee bioluminescence decreased following surgical destabilization and exercise. Surgically destabilized and exercised (SDE, n = 3) mice were trained for exercise prior to surgical destabilization of their left knees by transection of the medial collateral ligament and removal of the medial meniscus. After surgery and a 3-day rest period, SDE mice were subjected to daily running for 1 week. Control (CTRL, n = 3) mice were allowed unrestricted activity within their cages and handled identically to SDE mice without exercise or surgery. (A) Bioluminescence decreased with time post-destabilization for the SDE mice, but not for the control mice. (B) Quantitative bioluminescence images of a representative mouse.

To assess the utility of this mouse for quantifying cartilage during *in vivo* longitudinal studies, we performed bioluminescence imaging on live mice under anesthesia. The total pixel intensity decreased with the age of the mouse (p < 0.001). Signal from 2 month old mice was more than 3-fold greater than signal from 5-month and 13-month old mice (Fig 4A and 4B, n = 10 mice for each age). Histological examination (Fig 4C) found a decrease in articular cartilage thickness and loss of Toluidine Blue staining between 2- and 5-month old mice. There were fewer chondrocytes after 5 months, consistent with the decreased bioluminescence (Fig 4C). In a treadmill model of OA, there was a decrease in total pixel intensity with model progression (r = -0.526, p = 0.0028, Fig 5A). Histological evaluation found the cartilage layer disrupted after 54 days (Fig 5C). There was decreased toluidine blue staining, thinner cartilage, and loss of chondrocytes after intensive treadmill running which are hallmarks of OA. These changes correlated with the loss in bioluminescence (Fig 5B and 5C). For mice subjected to both surgical destabilization and exercise (SDE), we observed a negative correlation between time and bioluminescence beginning on day 8 which was not observed in the control mice (Fig 6, r_SDE_ = -0.973, p_SDE_ = 0.027. r_CTRL_ = 0.459, p_CTRL_ = 0.540).

**Fig 7:**
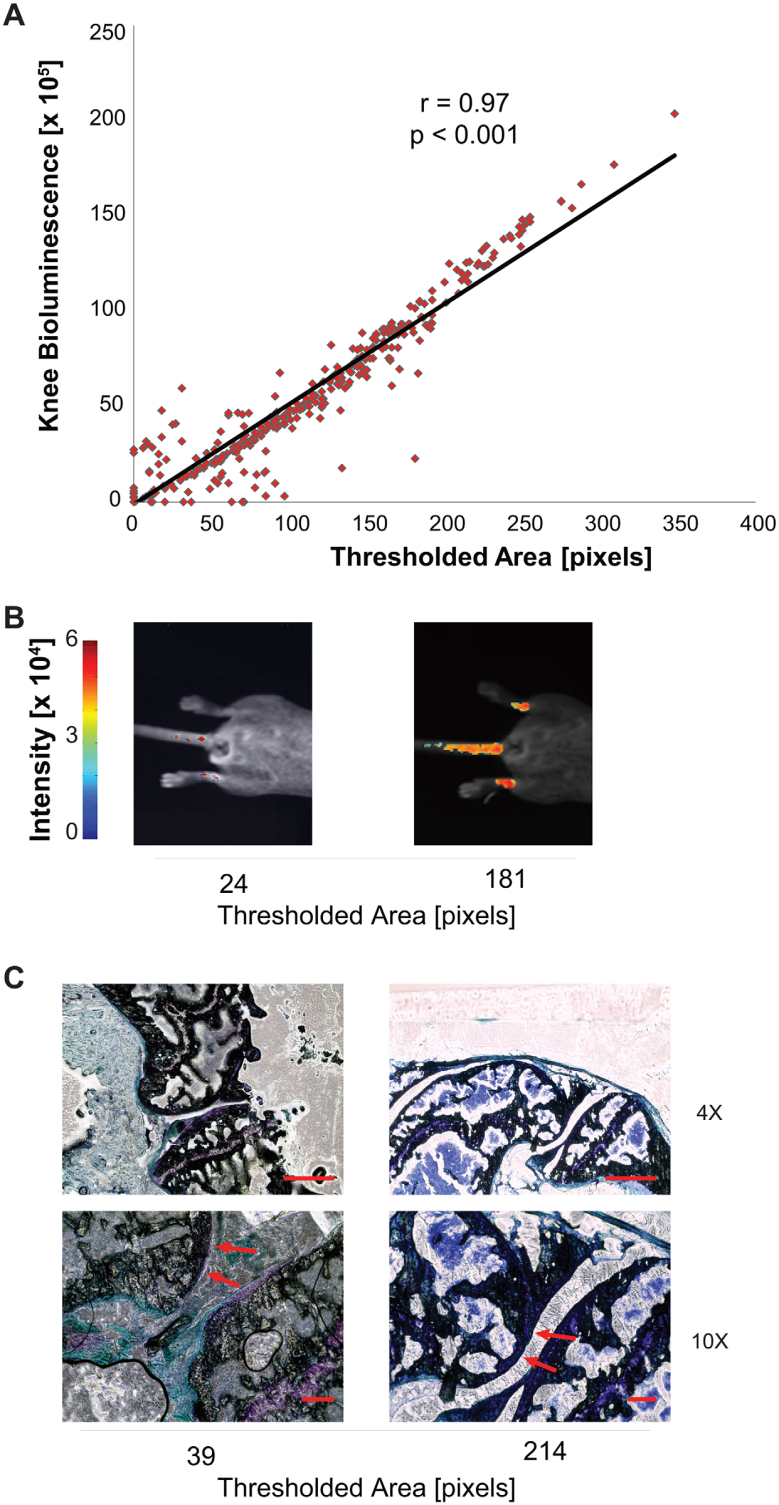
Quantitative relationship between pixel area and intensity over all images. (A) There is a linear correlation between pixel area and total pixel intensity (r = 0.9748, p < 10^-5^). (B,C) Representative quantitative bioluminescence images and histology from mice with low (Left) or high total pixel intensities. Low pixel intensities were defined as knee signal less than 5 x 10^6^ and high intensities were defined as signals greater than 10 x 10^6^. Qualitatively, the histology shows less cartilage in the low-signal images (left) and more cartilage in the high-signal images (right) indicating that knee bioluminescence in these transgenic mice may be a useful *in vivo* and longitudinal measure of cartilage to complement histopathological studies. Scale bars: (4X) 200 microns (10X) 50 microns.

We found a strong linear correlation between area and total pixel intensity (r = 0.9748, p < 0.001, n = 676 images, Fig 7). The bioluminescence measures were consistent with histological observations that found decreases in cartilage area in samples with decreased quantitative signal (Fig 7B and 7C). These results indicate that bioluminescence can be used as a non-invasive surrogate measure for the amount of cartilage within a joint during studies of experimental OA and aging.

## Discussion

In the study presented here, we demonstrate for the first time the ability to use a targeted insertion within a non-coding region of an endogenous, chondrocyte-specific gene (*i*.*e*. aggrecan) to drive chondrocyte-specific transcription of CreERT2 which activates luciferase expression for quantitative, non-invasive *in vivo* reporting in mouse joints. This system can complement existing histological and imaging approaches through non-invasive, quantitative, and longitudinal monitoring of cartilage as a proxy for OA progression during experimental murine osteoarthritis. This technology has the potential to advance the rate of both basic and translational science for preventing and repairing cartilage damage.

In this study, significant differences in cartilage-specific bioluminescence were obtained using group sizes of three to five mice (Figs 5 and 6). For comparison, other studies utilizing solely histopathological grading scales have required between 7 and 33 mice to reach significance [11,31–34]. The *in vivo* models found decreases in bioluminescent signal during their progression, indicating that this mouse system is compatible with common experimental models of murine OA, including surgical destabilization and forced high-intensity exercise. In the present model, there was negligible leakiness of the endogenous aggrecan promoter, as reported previously [21]. Using initial imaging parameters of a 15 minute exposure occurring 30 minutes after scruff injections, we found joint- and cartilage-associated bioluminescence in several conditions. As expected, lower bioluminescent signals were noisier and weaker, but we expect improved imaging instrumentation [35] will likely further enhance the results. In these studies, subcutaneous scruff injections provided the most consistent method of luciferin administration due to a reduction in error and variability as compared to local subcutaneous and intraperitoneal injection (data not shown). The challenge of luciferin delivery to chondrocytes in the articular cartilage in this mouse model likely represents the same drug delivery challenge for treating articular chondrocytes *in vivo* [2,36].

Tamoxifen, required to activate creERT2 activity, has the potential to alter skeletal biology by altering inflammation and other endogenous processes. Therefore, in this study we used a 7-day resting period between tamoxifen administration and imaging based on previous studies that used between 0 and 14 days [37–39]. The present histological processing methods were selected in an attempt to perform bioluminescence imaging on tissue sections which ultimately proved infeasible, and findings of this study would likely be improved using standard methods of fixation and decalcification. Comparison of the luminescent signals with measurements of cartilage volume (*e*.*g*. from microCT) would further relate the quantitative bioluminescent signal to a relevant variable regarding joint health.

The mouse model described here has many potential applications for both basic and pre-clinical OA research. The ability to time-stamp chondrocytes with a genetically encoded bioluminescent signal induced by tamoxifen for the first time enables *in vivo* longitudinal studies of chondrocyte death and proliferation. Future studies may combine bioluminescence measurements with *in situ* hybridization using probes to identify specific cells having the tamoxifen-induced time-stamp at experimentally relevant time points in various models (*e*.*g*. post-traumatic osteoarthritis). Utilization of the aggrecan creERT2 mouse in combination with floxed genes of interest in murine OA (*e*.*g*. MMP13 [33]) would result in a mouse with cartilage-specific inducible bioluminescence and the absence of the gene of interest specifically within cartilage, allowing researchers to study the timepoint of gene deletion during the time course of post-traumatic OA to define relevant therapeutic intervention frameworks for siRNA and other inhibitory therapeutics. Finally, chondrocyte drug-delivery can be effectively modeled using the Luc/Luc creERT2/creERT2 mouse. By examining the onset and duration of bioluminescence via luciferin administration, drug delivery technologies (*e*.*g*. nanoparticle encapsulation) may be assessed.

In summary, non-invasive live bioluminescence imaging revealed quantitative joint-specific differences in joint cartilage under following conditions of cartilage stress or deterioration. Enzymatic digestion experiments measured decreasing signal with increasing digestion. This mouse model provides a promising tool for both sensitive and longitudinal quantification of cartilage deterioration in experimental arthritis studies.

## Supporting Information

S1 Fig Comparison of 10 and 15 minutes of exposure time at 30 minutes after luciferin administration. 15 minutes resulted in ~3.5-fold stronger signal (p < 0.01).
(PDF)

S2 Fig Male and female mice exhibit indistinguishable bioluminescent signals (p = 0.998). (PDF)

S3 Fig Comparison of tamoxifen free base versus 4-OHT. Bioluminescent signal was not different between the types of tamoxifen (p = 0.83).
(PDF)

S4 Fig Examination of the imaging timepoint following luciferin administration. Symbols represent datapoints, and bars represent median values. No signal was detected prior to luciferin injection. We observed an increase in bioluminescent signal from 0 to 30 minutes (p = 0.018) and from 30 to 60 minutes (p = 0.012). The relative variability was larger 60 minutes after luciferin injection.
(PDF)

S5 Fig Effect of various physical and optical methods on maximizing the bioluminescence. Glycerine was applied topically around the knee joints to reduce light scatter. Physical filters made from opaque drinking straws were used to direct knee bioluminescence to the camera. There were no statistical differences between the control and either experimental group.
(PDF)
