# Supplementary figures and images for: Non-Invasive Quantification of Cartilage Using a Novel In Vivo Bioluminescent Reporter Mouse
Source: PLoS One. 2015 Jul 7;10(7):e0130564. doi: 10.1371/journal.pone.0130564 (PMC4495059; doi:10.1371/journal.pone.0130564)

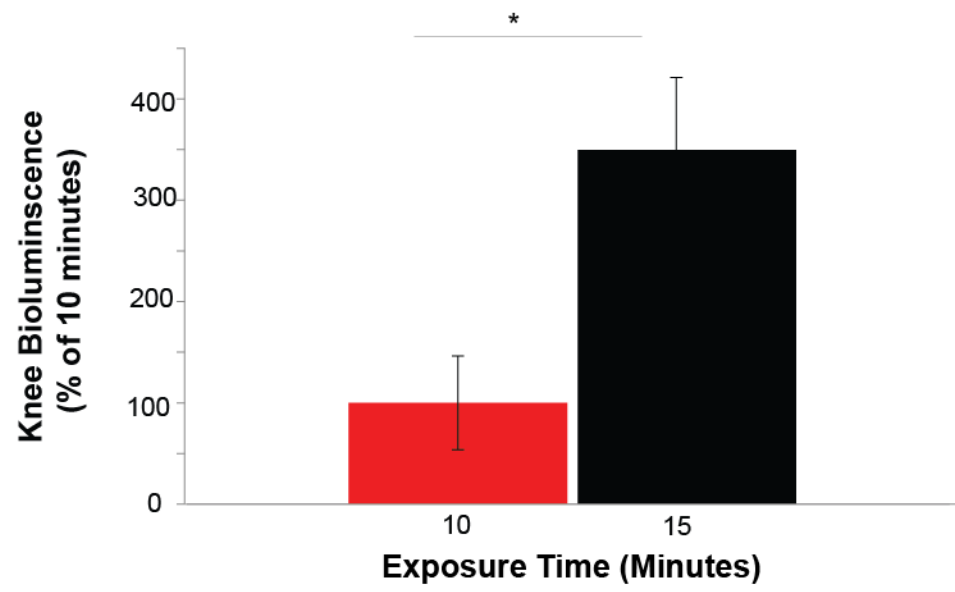

Supplement: S1 Fig — 15 minutes resulted in ~3.5-fold stronger signal (p < 0.01). (PDF) [file pone.0130564.s001.pdf]

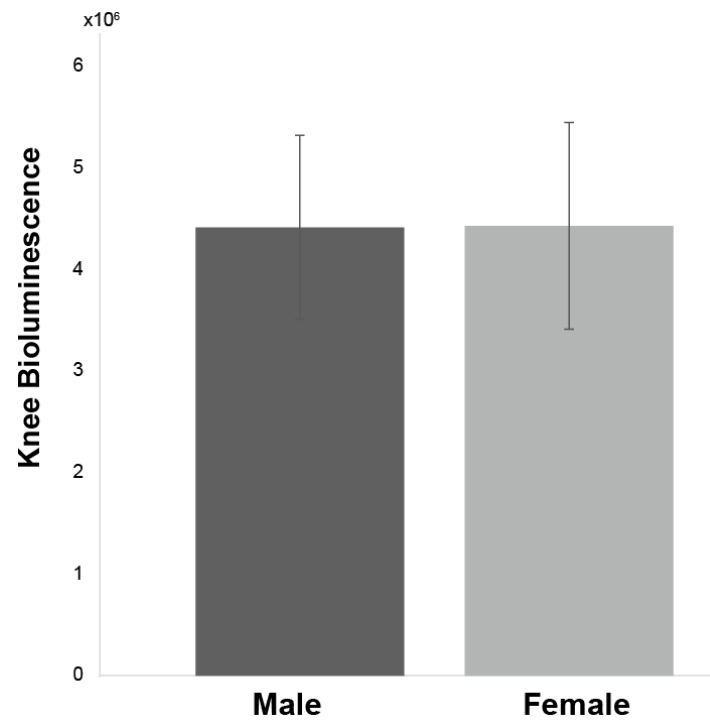

Supplement: S2 Fig — (PDF) [file pone.0130564.s002.pdf]

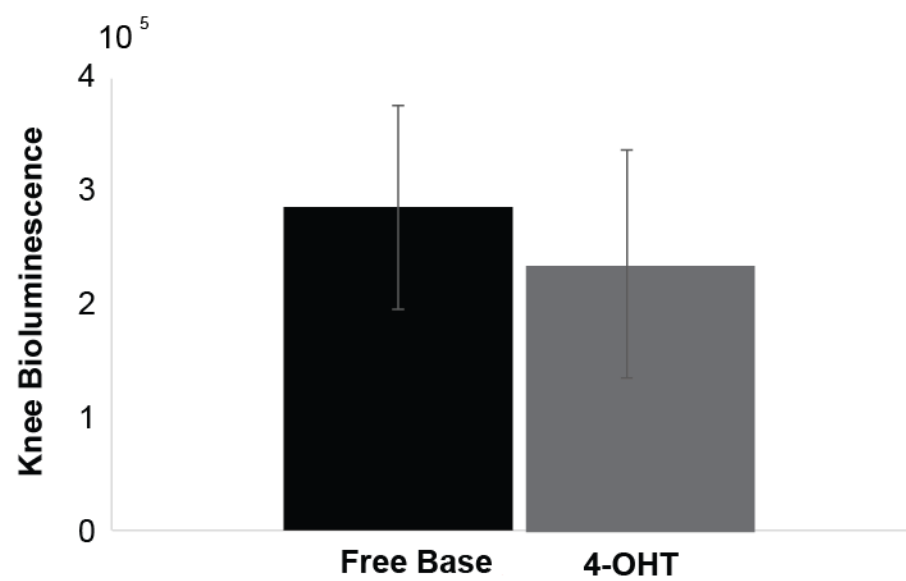

Supplement: S3 Fig — Bioluminescent signal was not different between the types of tamoxifen (p = 0.83). (PDF) [file pone.0130564.s003.pdf]

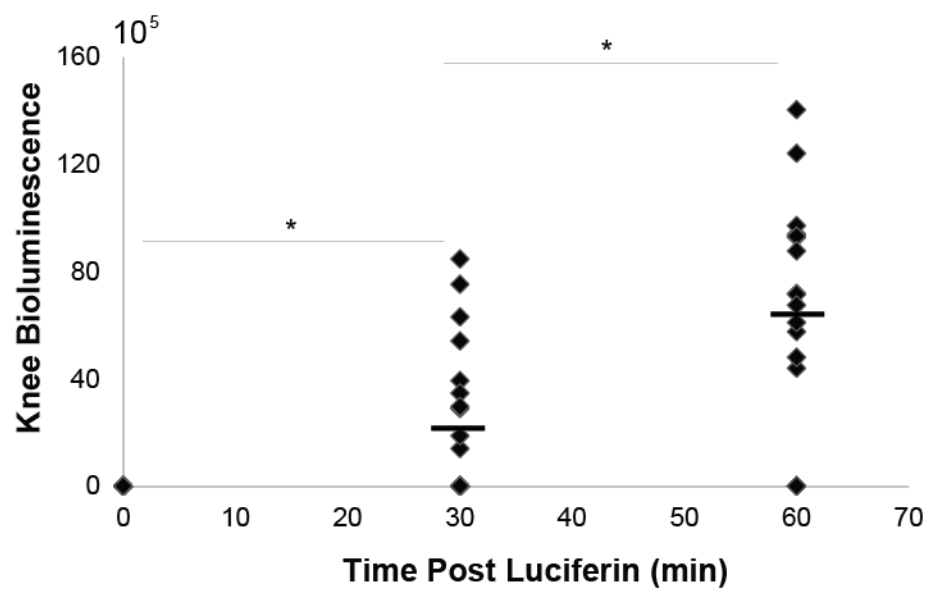

Supplement: S4 Fig — Symbols represent datapoints, and bars represent median values. No signal was detected prior to luciferin injection. We observed an increase in bioluminescent signal from 0 to 30 minutes (p = 0.018) and from 30 to 60 minutes (p = 0.012). The relative variability was larger 60 minutes after luciferin injection. (PDF) [file pone.0130564.s004.pdf]

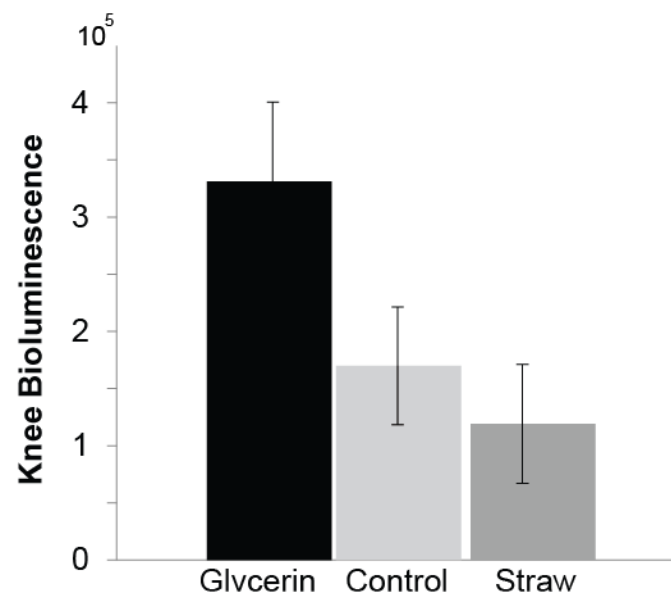

Supplement: S5 Fig — Glycerine was applied topically around the knee joints to reduce light scatter. Physical filters made from opaque drinking straws were used to direct knee bioluminescence to the camera. There were no statistical differences between the control and either experimental group. (PDF) [file pone.0130564.s005.pdf]
